# Data supporting the short-term health effects of temperature and air pollution in Valencia, Spain

**DOI:** 10.1016/j.dib.2022.108518

**Published:** 2022-08-05

**Authors:** Carmen Iñiguez, Ferran Ballester, Aurelio Tobias

**Affiliations:** a Department of Statistics and Operational Research, Universitat de València, Calle Dr Moliner 50, València, Burjassot 46100, Spain; b Department of Nursing, Universitat de València, Valencia, Spain; c CIBER de Epidemiología y Salud Pública (CIBERESP), Spain; d Epidemiology and Environmental Health Joint Research Unit, FISABIO- Universitat Jaume I- Universitat de València, Valencia, Spain; e Institute of Environmental Assessment and Water Research (IDAEA), Spanish Council for Scientific Research (CSIC), Barcelona, Spain; f School of Tropical Medicine and Global Health, Nagasaki University, Nagasaki, Japan

**Keywords:** Environmental health, Short-term effects, Mortality, Temperature, Air pollution, Time-series, Poisson regression, Distributed lag non-linear models

## Abstract

The data presented in this article is part in essence of a more extensive dataset aimed at evaluating patterns of change in the temperature–mortality relationship on population health in the city of Valencia, Spain on population health in the city of Valencia, Spain. The complete dataset was used in the framework of the European multi-city project PHASE (Public Health Adaptation Strategies to Extreme weather events) [1]. The data includes daily counts of all-cause mortality, excluding external causes and cardiovascular and respiratory diseases. All-cause mortality is also classified by gender and age groups. Besides temperature, we included other meteorological variables and air pollutants from the PHASE dataset, as well as influenza epidemics. The variable Saharan dust events was also added. All these data were collected from public Governmental data repositories accessible under request. The dataset of this article provides a basis for comparison with similar models for time-series regression, allowing researchers to integrate additional model components without duplication of effort.

## Specifications Table

**Table:** 

Subject	Environmental health
Specific subject area	Short-term health effects of environmental risk exposures
Type of data	Graphs, figures and tables
How data were acquired	All variables were gathered from public Governmental data repositories accessible under request
Data format	Raw and analysed
Parameters for data collection	All the variables were collected on a daily basis for the study period between 1st January 2001 and 31st December 2007. Daily counts of all-cause mortality data were collected by gender and age groups (< 15 years, 15–64 years, ≥ 65 years). Meteorological variables and air pollutants concentrations were collected as daily averages
Description of data collection	The authors collected data from public Governmental data repositories accessible under request to be used in the PHASE project [1]
Data source location	City of Valencia, Spain
Data accessibility	Repository Name: ValenciaTempMortDirect URLs to the data: https://data.mendeley.com/datasets/2cxkjjhrnf/
Related research article	de' Donato FK, Leone M, Scortichini M, De Sario M, Katsouyanni K, Lanki T, Basagaña X, Ballester F, Åström C, Paldy A, Pascal M, Gasparrini A, Menne B, Michelozzi P. Changes in the Effect of Heat on Mortality in the Last 20 Years in Nine European Cities. Results from the PHASE Project. Int J Environ Res Public Health. 2015; 12(12): 15,567–83. doi:10.3390/ijerph121215006

## Value of the Data

• This dataset allows the estimation of short-term health effects of meteorological variables and air pollutants, and assess the impact of environmental policies on population health.
• These data can be used for educational purposes to illustrate the use of time-series regression and distributed lag non-linear models in environmental epidemiology studies.
• These data provide a basis for comparison with similar models and allow researchers to integrate additional model components without duplication of effort.

## 1 Data

### 1.1 Description of Study Area

The city of Valencia, the capital of the Spanish province of the same name, is located on the eastern coast of the Iberian Peninsula and the western part of the Mediterranean Sea (latitude 39° 28 N, longitude 0° 22 W). Spreading on an area of 135 km2, Spain's third-most populated municipality, with 789,744 inhabitants.

According to the Köppen climate classification (Iberian Climate Atlas (2011)), Valencia has a Hot-summer Mediterranean climate (csa category) with mild winters and hot, dry summers.

### 1.2 Mortality Data

Mortality data are represented by daily counts for all causes, excluding external causes (natural mortality, International Classification of Diseases, 9th and 10th Revisions, ICD-9: 1–799 and ICD-10: A00-R99), cardiovascular diseases (ICD-9: 390–459, ICD-10: I00-I99) and respiratory diseases (ICD-9: 460–519, ICD-10: J00-J99). Mortality counts for all causes were classified by gender and in three age groups (< 15 years, 15–64 years, ≥ 65 years), commonly used in environmental epidemiology to identify vulnerable age groups. Data were obtained from the Valencian community mortality register [1]. All series of mortality counts were complete (no missing values).

### 1.3 Meteorological Data

Daily mean, minimum and maximum temperature (°C) and relative humidity (%) were collected from the National Institute of Meteorology at one city's weather station located in the Meteorological Centre of Valencia [1]. We also identified heatwave days defined as those periods of at least two days with maximum temperature exceeding the 90th percentile of the monthly distribution between May and October, or those periods of at least two days in which the minimum temperature exceeds the 90th percentile of the monthly distribution and the maximum temperature exceeds the median monthly value [2]. All series of meteorological data were complete (no missing values).

### 1.4 Air Pollution Data

Daily particulate matter with aerodynamic diameter ≤ 10 µm (PM10, 24 h average), nitrogen dioxide (NO2, 24 h average), and ozone (O3, maximum 8 h moving average) were collected from the Valencian community's Air Pollution Monitoring Network [1]. A conversion factor was used to obtain estimates of PM10 measurements from total suspended particles (TSP), as PM10 = TSP × 0.58 [3]. The number of missing values were *n* = 282 (11%), 158 (6.2%) and 88 (3.4%) for PM10, NO2 and ozone, respectively. We also collected those days with Saharan dust intrusions from the Spanish Ministry for the Ecological Transition (Ministerio para la Transición Ecológica, MITECO). It is based on the daily interpretation of air mass back trajectories, synoptic meteorological charts, satellite imagery and daily consultation of dust forecast models [4]. However, the identification of Saharan dust in Spain was established in 2003. Since this year, the series has been completed (no missing values).

### 1.5 Other Data

The dataset also includes an indicator variable for influenza epidemics, complete along the period (no missing values), obtained from the epidemiological services of the city of Valencia [5], and calendar variables for date, year, month, day of the month, day of the week and public holidays.

## 2 Experimental Design, Materials and Methods

**Fig. 1:**
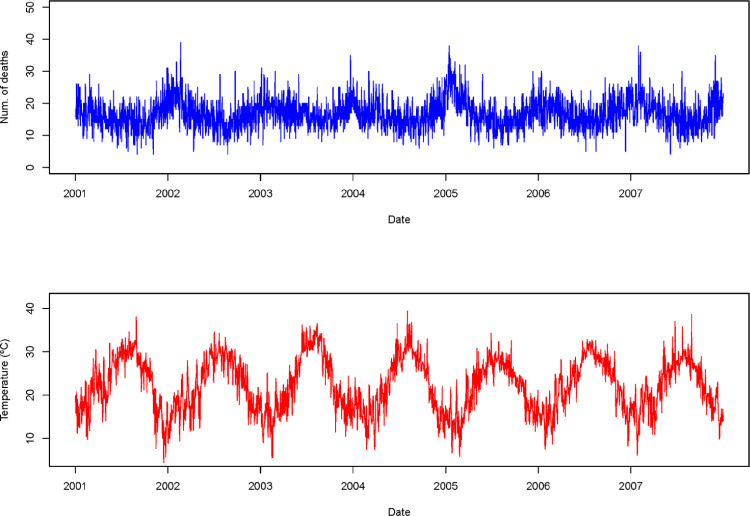
Daily counts of all-cause mortality and mean temperature ( °C) in Valencia, Spain, 2001–2007.

The dataset has been used to evaluate the association between temperature and mortality using time-series regression [1]. Here, exposure and outcome data are available at regular time intervals (i.e. daily temperature and mortality counts) [6]. The time-series design has been widely used in environmental epidemiology to investigate short-term associations between environmental exposures such as air pollution, weather variables or pollen, and health outcomes such as mortality or disease-specific hospital admissions [7]. In this illustration, we analysed the association between daily mean temperature and mortality. Fig. 1 shows the seasonal patterns for daily temperature and mortality over time.

Data is analysed using quasi-Poisson regression with distributed lag non-linear models (DLNM) [8]. This class of models can describe complex non-linear and lagged dependencies by combining two functions that define the conventional exposure-response association and the additional lag-response association, respectively. The lag-response association represents the temporal change in risk after a specific exposure, and it estimates the distribution of immediate and delayed effects cumulated across the lag period.

Specifically, we modelled the temperature-mortality relationship using a natural cubic spline, with three internal knots at the 10th, 75th, and 90th percentiles of the temperature distribution, and the lag-response relationship using a natural cubic spline with three internal knots equally spaced on the logarithmic scale. The lag period was extended to 21 to capture the long delay in the effects of cold. The model also included a natural cubic spline of time with 10 degrees of freedom per year to control seasonal variations and long-term trends and indicator variables for days of the week and public holidays. These modelling choices are based on the previously extensive work using an overlapping dataset and have been thoroughly tested by sensitivity analyses [9], [10], [11].

Statistical analysis was performed in R software, version 4.1.1, using the library DLNM [12]. The *R* code to analyse the data is available in the Appendix as Supplementary data.

**Fig. 2:**
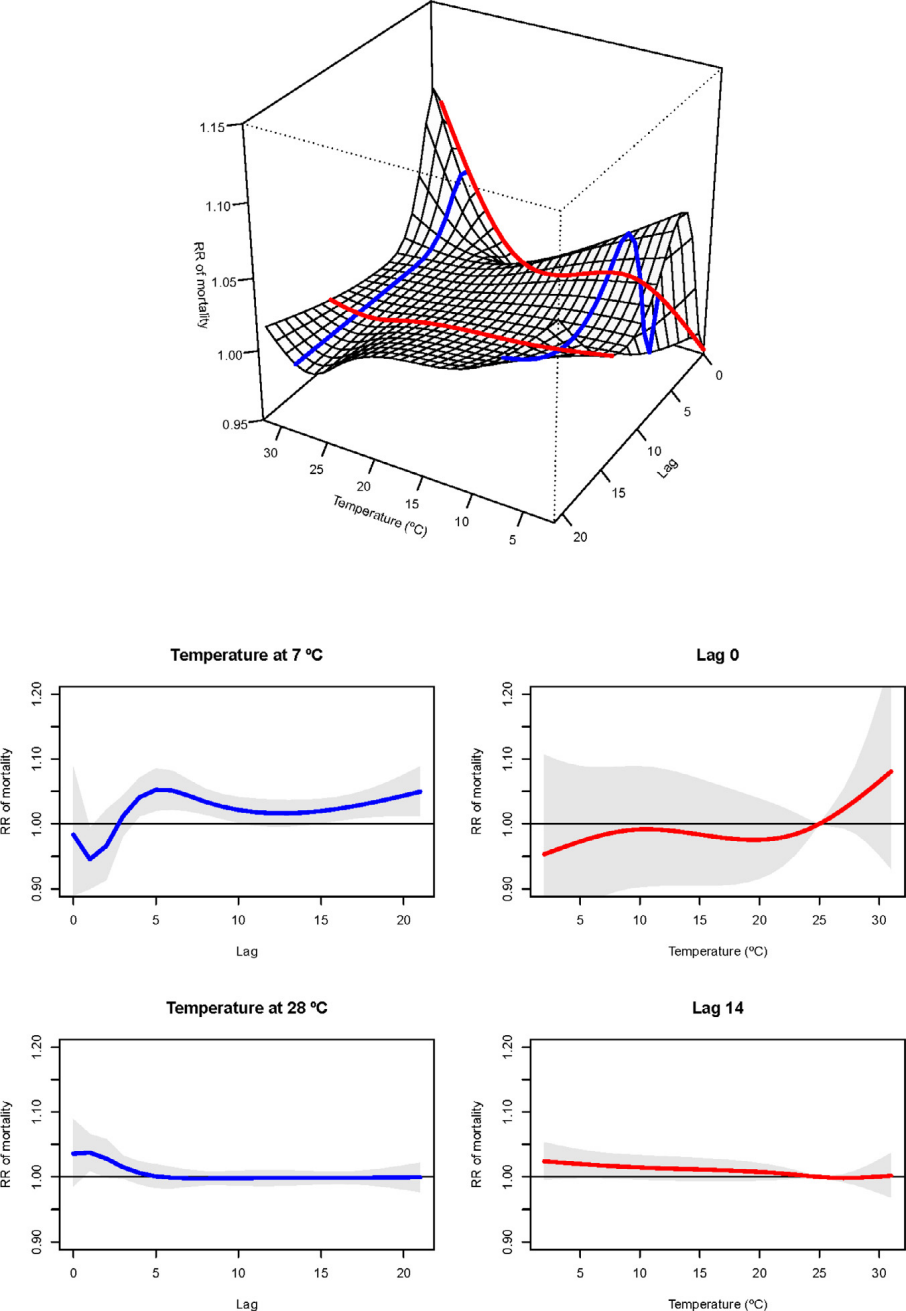
Relative risk (RR) of mortality along with daily temperature and lag dimension with reference at 25 °C (top panel); plot of RR by cold and heat temperatures (7 °C and 28 °C, respectively) at specific lags (bottom-left panel); and plot of RR at lags 0 and 14 along with temperature distribution (bottom-right panel).

**Fig. 3:**
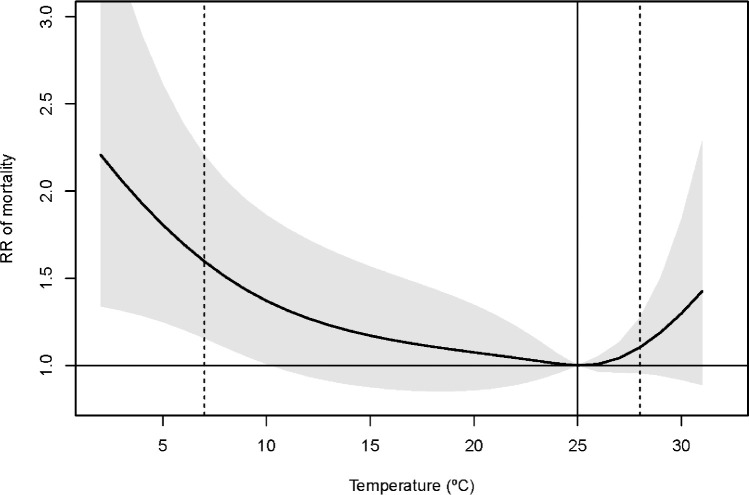
Overall cumulative exposure-response association between daily temperature and mortality across all lags, with related temperature distribution. The solid vertical line is the minimum mortality temperature at 25 °C, and dashed vertical lines are the 2.5th and 97.5th percentiles of the temperature distribution at 7 °C and 28 °C, respectively.

The specification of a DLNM implies a complex parametrization of the exposure series relying on a set of coefficients which straightforward estimation (common regression) but with no straightforward interpretation. The library DLNM, helps the user by providing him with the interpretation of these coefficients in terms of a surface of estimated effects along the two dimensions (object *crosspred*) and robust graphical capabilities (*plot.crosspred*), allowing for interesting summaries, as illustrated in Figs. 2 and 3.

Fig. 2 shows the relative risk (RR) of mortality along with daily ambient temperature and lag dimension. The RR is interpreted as the ratio between the risk of a specific temperature at a specific lag compared with the risk at the temperature at which the risk of mortality is minimum (MMT) (9), located at 25 °C. The blue lines show the RR across the lag dimension for the cold and heat effects defined at the 2.5th and 97.5th percentile of the temperature distribution, located at 7 °C and 28 °C, respectively. In contrast, the red lines indicate the RR at lags 0 and 14 days along with temperature distribution (i.e., the same day when the temperature exposure occurs and one week after the exposure). Fig. 3 shows the cumulative exposure-response association between daily temperature and mortality across all lags, with related temperature distribution. Again, the cold effect is defined as the risk of mortality at the 2.5th percentile of the temperature distribution (RR = 1.51, 95%CI = [1.11, 2.07]) and the heat effect at the 97.5th percentile (RR = 1.19, 95%CI = [0.94, 1.51]).

## Ethics Statements

The authors declare that there are no ethical issues with data and methods used in this research and that these data are neither involved with human subjects, animal experiments nor obtained from social media platforms.

## CRediT authorship contribution statement

**Carmen Iñiguez:** Conceptualization, Data curation, Software, Methodology, Writing – review & editing. **Ferran Ballester:** Data curation, Writing – review & editing. **Aurelio Tobias:** Conceptualization, Writing – original draft, Methodology.

## Declaration of Competing Interest

None.

## Data Availability

DBval (Reference data) (Mendeley Data).
